# The extraction of the new components from electrogastrogram (EGG), using both adaptive filtering and electrocardiographic (ECG) derived respiration signal

**DOI:** 10.1186/s12938-015-0054-0

**Published:** 2015-06-23

**Authors:** Dariusz Komorowski, Stanislaw Pietraszek, Ewaryst Tkacz, Ivo Provaznik

**Affiliations:** Department of Biosensors and Biomedical Signals Processing, Faculty of Biomedical Engineering, Silesian University of Technology, 40 Roosevelt’a Street, 44-800 Zabrze, Poland; Division of Biomedical Electronics, Institute of Electronics, Silesian University of Technology, 16 Akademicka Street, 44-100 Gliwice, Poland; Department of Biomedical Engineering, Brno University of Technology, 12 Technicka Street, 61200 Brno, Czech Republic; International Clinical Research Center, Center of Biomedical Engineering, St. Anne’s University Hospital Brno, Brno, Czech Republic

**Keywords:** Electrogastrography, Electrogastrogram, Electrocardiographic derived respiration signal, Adaptive filters, QRS detection

## Abstract

Electrogastrographic examination (EGG) is a noninvasive method for an investigation of a stomach slow wave propagation. The typical range of frequency for EGG signal is from 0.015 to 0.15 Hz or (0.015–0.3 Hz) and the signal usually is captured with sampling frequency not exceeding 4 Hz. In this paper a new approach of method for recording the EGG signals with high sampling frequency (200 Hz) is proposed. High sampling frequency allows collection of signal, which includes not only EGG component but also signal from other organs of the digestive system such as the duodenum, colon as well as signal connected with respiratory movements and finally electrocardiographic signal (ECG). The presented method allows improve the quality of analysis of EGG signals by better suppress respiratory disturbance and extract new components from high sampling electrogastrographic signals (HSEGG) obtained from abdomen surface. The source of the required new signal components can be inner organs such as the duodenum and colon. One of the main problems that appear during analysis the EGG signals and extracting signal components from inner organs is how to suppress the respiratory components. In this work an adaptive filtering method that requires a reference signal is proposed. In the present research, the respiratory component is obtained from non standard ECG (NSECG) signal. For purposes of this paper non standard ECG (namely NSECG) is used, because ECG signal was recorded by other than the standard electrodes placement on the surface of the abdomen. The electrocardiographic derived respiration signal (EDR) is extracted using the phenomena of QRS complexes amplitude modulation by respiratory movements. The main idea of extracting the EDR signal from electrocardiographic signal is to obtain the modulating signal. Adaptive filtering is done in the discrete cosine transform domain. Next the resampled HSEGG signal with attenuated respiratory components is low pass filtered and as a result the extended electrogastrographic signals, included EGG signal and components from other inner organs of digestive system is obtained. One of additional features of the proposed method is a possibility to obtain simultaneously recorded signals, such as: non-standard derivation of ECG, heart rate variability signal, respiratory signal, and EGG signal that allow investigating mutual interferences among internal human systems.

## Background

Electrogastrography (EGG) is a technique for non-invasive recording of gastric myoelectrical activity [1–4]. The multichannel classic surface EGG signals are captured by six disposable electrodes placed on the anterior abdominal wall overlying the stomach. This technique can be considered as a non-invasive method for investigating the propagation of slow waves in the stomach. Their normal frequency is about three cycles per minute (cpm) (0.05 Hz) in humans. The EGG examination may be helpful in diagnosis of gastric disorders. It could diagnose the patients with unexplained nausea, vomiting and other dyspeptic symptoms [1]. Typically, the EGG signals are collected during relatively long time (120–180 min), and the examination is split into three parts: the first a 30-min part before meal (pre-prandial), the second (5–10 min)—during a standardized meal, and the third one after the meal (postprandial). The EGG signals are characterized by following parameters: frequency ranges from 0.015 to 0.15 Hz and maximum amplitude is 500 μV. Usually in clinical applications the acquisition process is performed by commercial devices with relatively low sampling frequency (1–4 Hz) and EGG signals are conditioned by means of proper band pass filtering [4]. The detail description of both EGG processing methods and its diagnostic significance is available in Ref. [5, 6].

Besides EGG, also other signals are available on the stomach surface. They are related to electrical activity of other inner organs of the digestive system such as the duodenum and colon, to heart activity and respiratory movements. The frequency components of these signals partially cover frequency range of EGG signals.

Commonly used conventional band pass filtering may cause loss of some part of information included in this signal especially signals from duodenum and colon or may introduce distortions of EGG signal. In this work a new method for extracting signal components of inner organs of digestive system from the high sampling frequency electrogastrographic signal (HSEGG) is proposed. One of the most important problems is dumping the respiratory components in recorded signal [7]. In this work, an adaptive filtering was used. It is very useful for attenuation of the superfluous signals but requires the reference signal. In our case source of this signal comes from electrocardiographic derived respiration signal (EDR)—respiratory signal derived from electrocardiographic signal (ECG). The method of extracting respiratory signal uses the effect of modulation of some parameters of ECG signal by respiratory movements [8]. The most frequently used parameters are: amplitude of R wave and area under QRS complex. The main idea of EDR methods is to reconstruct the modulating signal. Nowadays these methods are widely introduced to clinical practice because they reduce the number of sensors or equipment. In this work, we propose to record signals with relatively higher sampling frequency (i.e. 200 Hz) than it is typically used for acquisition of electrogastrographic (EGG) signals (1–4 Hz) [4]. This high frequency allows to record extended EGG components. The EDR signal is evaluated from HSEGG signal and then used as a reference signal for adaptive filtering. The adaptive filtering of resampled to 4 Hz HSEGG signal is performed in the discrete cosine transform (DCT) domain. Next the signal with attenuated respiratory components is band pass filtered. As a result, the extended electrogastrographic signals **(**ExEGG) signals are obtained. In this work the EDR signals is reconstructed by analysis of the R wave amplitude. Changes of the R wave amplitude are estimated by means of calculating the area under QRS complexes. The method of estimation of EDR has been chosen in the presented way due to the fact that other approach, like for example changes of heart electrical axes against the time, would require application of classically registered ECG signal i.e. with the application of standard ECG leads. Recently more attention is paid to mutual interactions of different systems of the human body, such as digestive, cardiovascular, respiratory, and neural systems. A good example may be an influence of respiration, regulation of blood pressure or body temperature on the heart rate. The proposed method allows obtaining simultaneously recorded signals, such as: EGG, ECG (HRV), and respiration signal and use them to examine mutual interaction without any additional sensors and devices. A primary goal of this study was to improve the method of new signal components acquisition from stomach surface and to show that during EGG examination simultaneous acquisitions of other signals, such as heart rate variability **(**HRV) and EDR are possible.

## Methods

### HSEGG acquisition

**Figure 1:**
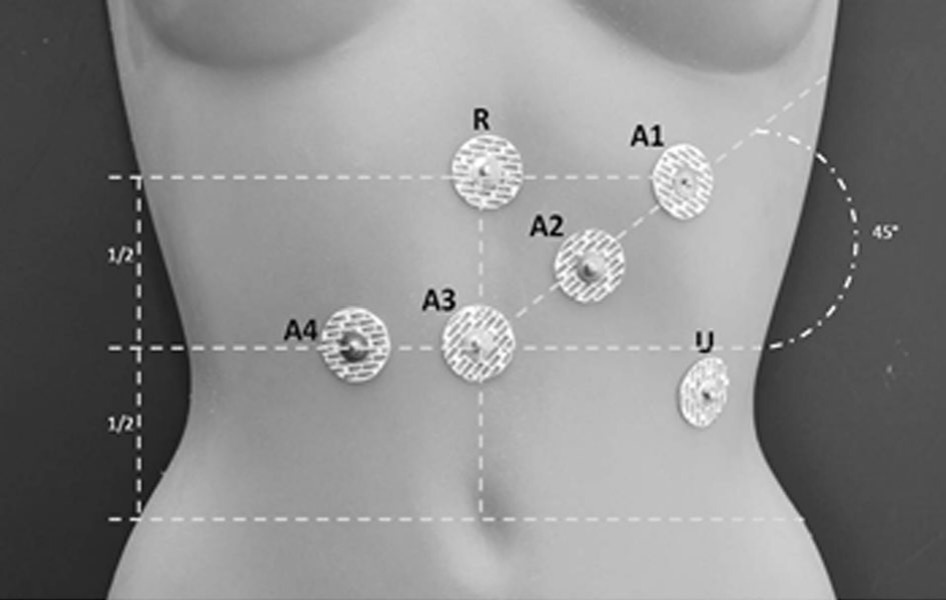
The standard placement of the EGG electrodes.

The HSEGG signals were recorded at the Department of Clinical Sciences of the Medical University of Silesia by means of the four-channel amplifier with galvanic isolation of patient’s side and following parameters: frequency band 0.015–50 Hz, gain 5,000, signal amplitude range ±2 mV, resolution—12 bits, and sampling rate 200 Hz per channel. Disposable EGG electrodes were applied according to the standard [4] during the signal registration process: four signal electrodes (A1–A4), reference electrode (R), and ground electrode (U) were included. An example of the electrodes placement is shown in Figure 1.

**Figure 2:**
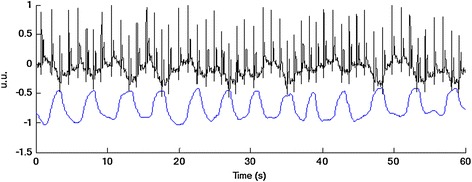
Examples of recorded signals (1 min). Normalized HSEGG signal after filtering with the fourth-order low pass Butterworth filter with a cutoff frequency of 35 Hz (*top*, *black line*). Normalized respiratory signal from thermistor after filtering with the fourth-order low pass Butterworth filter with a cutoff frequency of 0.5 Hz (*bottom*, *blue line*).

The additional respiratory signal was recorded by means of thermistor sensor (TDR, thermistor derived respiration) placed in front of the nose and the mouth of subject. This signal has been registered due to the need of comparison or better verification of respiratory signal obtained as EDR. It has been simultaneously acquired with the HSEGG signal and sampled with the same frequency equal to 200 Hz. The duration of records was 120–180 min. The examples of 1-min HSEGG signal (channel A2) and TDR signal are shown in Figure 2. The amplitudes of these signals are normalized to ±1, and to improve visibility, the curve of TDR in Figure 2 is shifted.

**Figure 3:**
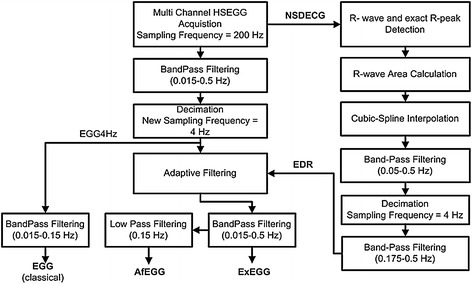
The block diagram of signals processing.

Next, the recorded signals were conditioned offline in MATLAB environment. In Figure 3, the block diagram of proposed algorithm is presented.

### Respiration signal extraction algorithm

During respiration, as a result of respiratory movements many parameters of ECG signal are modulated, e.g. amplitude of the ECG, heart rate (HR), and base line wandering [8–10]. In the presented work, the EDR signal is reconstructed by means of analyzing the modulation phenomena of R wave amplitude in the ECG. The respiration signal was evaluated by processing the area under QRS complexes in the ECG signal [11, 12].

**Figure 4:**
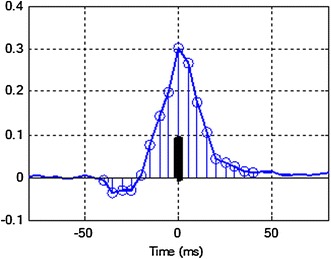
The method of *a*(*j*) parameter evaluation of the QRS complex. The *black vertical line* is an example of normalized area under QRS complex (*a(j)*) calculated according to formula (1).

**Figure 5:**
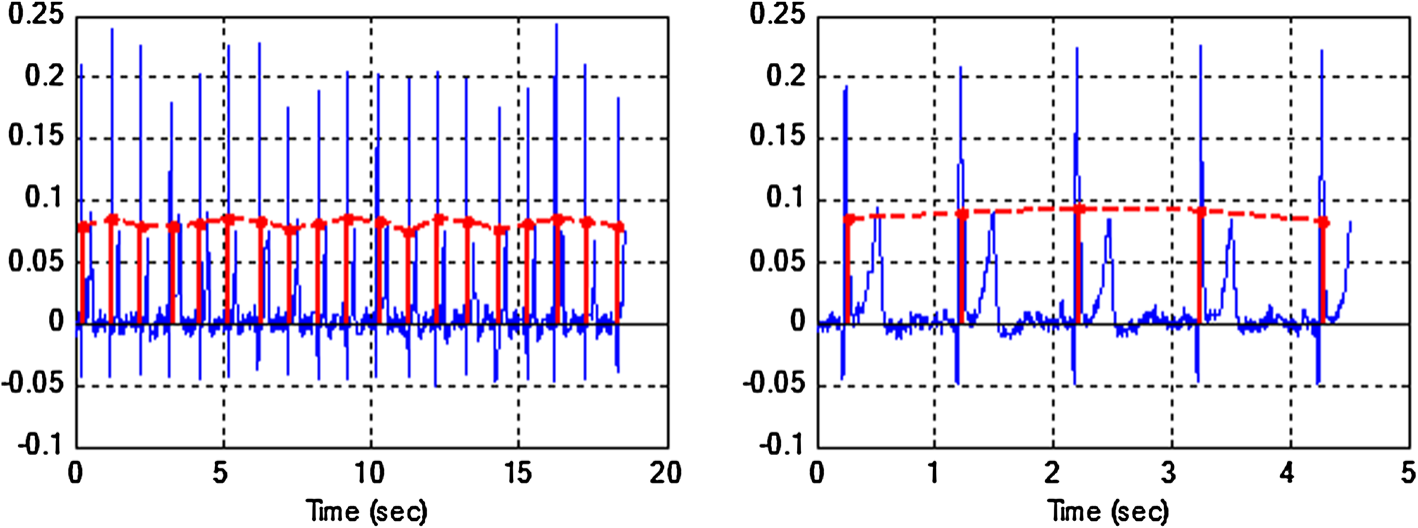
Interpolation of respiratory signal EDR (*left*) and its zoom (*right*). The *red vertical lines* indicate area under QRS complex (*a*(*j*)) calculated according to formula (1). The *dashed line* is interpolation of respiratory signal EDR.

If it is assumed that: N is the number of samples of HSEGG signal, *u*(*k*) is *k*-th sample of signal, $k = 1,2, \ldots ,{\text{N}}$ and peak *R* is *j*-th sample, the index of the area value for *j*-th QRS complex may be evaluated as:

1 $$a(j) = \frac{1}{2n + 1}\sum\limits_{k = j - n}^{j + n} {\left| {u(k)} \right|,}$$

where, *n* = 0.5*T*_*w*_*F*_*s*_, *T*_*w*_ is the window length for QRS area calculating and *F*_*s*_ is the sampling frequency. Respiratory signal EDR is evaluated by the interpolation of *a*(*j*) [sampled with 200 Hz (as the HSEGG) and decimated to 4 Hz (to avoid irregular sampling)]. The graphical illustrations of *a*(*j*) evaluation and interpolation of respiratory signal EDR are presented in Figures 4 and 5.

In the presented method, a detection of the QRS complexes has been done [13], the time instant of the R peak was found and value *a*(*j*) of index area for each QRS complex was calculated. The window symmetrically placed around R peak, with constant width of 80 ms [14] was used for *a*(*j*) calculating (Figure 4).

To improve the robustness to power noise that may appear in the signal, the width of window was chosen as a multiple of period of power line signal (20 ms). Analysis of reconstructed signal EDR shows that components connected with respiratory movements are present, while components connected with electrographic signal are not observed. So this signal may be used as a reference signal in adaptive filtering.

### Attenuating respiratory disturbances in EGG

**Figure 6:**
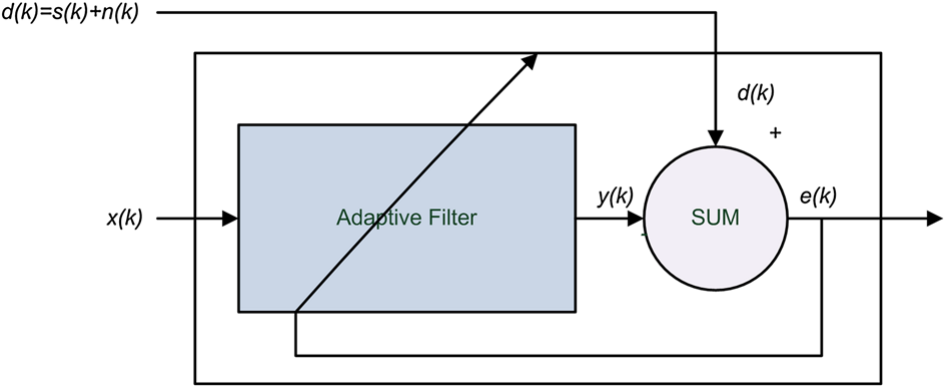
Using an adaptive filter to remove noise from an unknown system.

One of the methods used for improving the quality of recorded electrogastric signal is an application of adaptive filtering. This method is particularly useful for damping noise connected with respiratory movements in recorded signals. Because the frequency ranges of signals partially overlap, conventional band pass filtering may cause distortion in electrogastric signal. The standard method for attenuation of superfluous signal from recorded signal by means of adaptive filtering is shown in Figure 6.

In this figure, *d*(*k*) = *s*(*k*) + *n*(*k*) is the recorded signal after removing the unwanted fast components, *s*(*k*) is the desired signal, *n*(*k*) is the noise signal, *x*(*k*) is the reference signal correlated with noise signal *n*(*k*). Reference noise signal *x*(*k*) undergoes adaptive filtering for obtaining maximum correlation with input signal *d*(*k*). Because *s*(*k*) and *x*(*k*) are both generated by different sources, they are not correlated. Thus adaptive filter tune the signal *x*(*k*) to *n*(*k*) signal. In the output of adaptive filter the estimate $$y(k) = H(n(k)) = \overset{\lower0.5em\hbox{$\smash{\scriptscriptstyle\frown}$}}{n} (k)$$ for *n*(*k*) signal is obtained. Next, taking the equation $$e(k) = (d(k) - y(k)) = s(k) + (n(k) - \overset{\lower0.5em\hbox{$\smash{\scriptscriptstyle\frown}$}}{n} (k))$$ into consideration, signal *e*(*k*) is the desired signal without noise.

Usually adaptive filtering requires recording the reference signal that in case of biomedical signals may be difficult because of presence of noise with unknown characteristics. In some cases as the reference signal the modified and delayed original input signals are used. Adaptive filtering of EGG signal was proposed by Chen [15]. In his work the signal captured from another electrode was used as the reference signal. This signal was processed by the conventional band pass filtering and next was used as the reference signal for FIR adaptive filter. Another solution was presented by Liang [16]—the reference signal was obtained by means of principal component analysis of EGG signal. In the present work, the use of the EDR signal as the reference signal for adaptive filtering was proposed. In the first step the EDR signal has been band pass filtered in the range (0.2–0.5 Hz) [12], and as the result the signal components connected with respiratory movements have been obtained.

**Figure 7:**
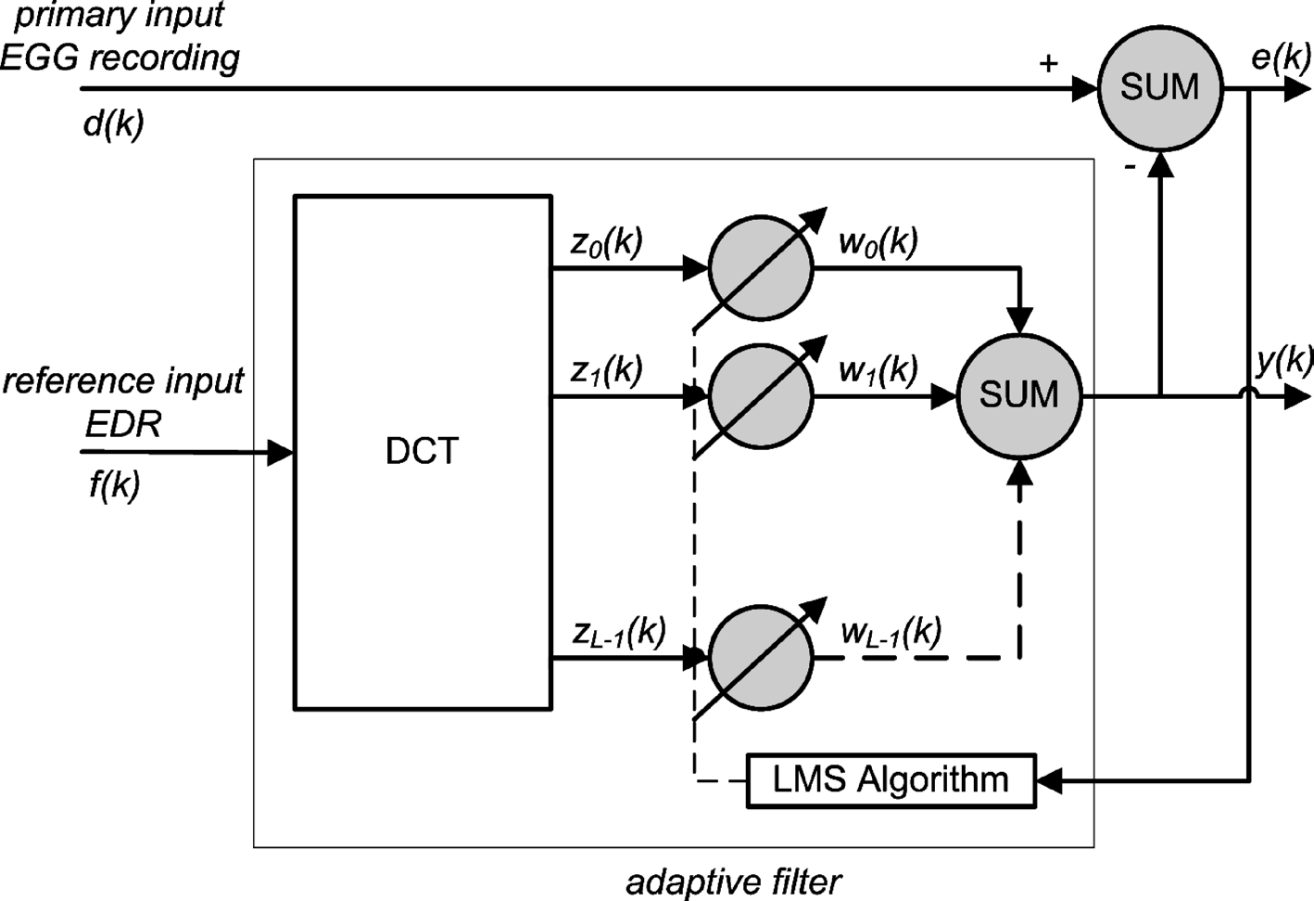
The *block diagram* of adaptive EGG signal processing using discrete cosine transform (DCT).

In the next step this signal is used as the reference signal for off-line implemented adaptive filtering. Adaptive filtering was performed in the transform domain, using the DCT. There are many solutions available in term of application of proper transform. However, an application of DCT transform in adaptive filter seems to be a compromise between convenience of implementation and achievable good results of EGG signals processing [16]. The least mean square algorithm (LMS) was used to update the weight coefficients [17, 18]. The block diagram of adaptive EGG signal processing using DCT is shown in Figure 7.

If *N* is the number of signal samples, *L* is the length of adaptive filter (number of weight coefficients), $k = 1,2, \ldots ,N - L + 1$ and if the mean square error:

2 $$mse = \frac{1}{N - L}\sum\limits_{k - 1}^{N - L} {e(k)^{2} }$$

for *e*(*k*) = *d*(*k*) − *y*(*k*) is defined as a difference between the filter output *y*(*k*) and input signal *d*(*k*) reaches minimum value, then the output signal:

3 $$Y = [y(1),\,y(2), \ldots y(N - L)]$$

is the best estimate (in the least square sense) for disturbing signal. The error signal:

4 $$E = [e(1),\,e(2), \ldots e(N - L)]$$

is the best (in the least square sense) estimate of the electrogastrographic signal as the input signal.

‘In other words, the adaptive filter produces a replica of that part of the primary input that is correlated with the reference input. The more the reference input contains correlated respiratory signal components, the better the system performance will be’ Liang [16, p. 247].

If the vector of reference signal *F*(*k*) is defined as:

5 $$F(k) = [f(k),f(k - 1), \ldots ,f(k - L)]$$

so the DCT of this vector is given by the equation:

6 $$Z(k) = [z_{1} (k),z_{2} (k), \ldots ,z{}_{L - 1}(k)]$$

and the filter weights are defined by the equation:

7 $$W(k) = [w_{0} (k),w_{1} (k), \ldots ,w_{L - 1} (k)]$$

the output of the adaptive filter may be described as follows:

8 $$y(k) = Z^{T} (k)W(k)$$

Values of weight coefficients in LMS algorithm are calculated according to formula:

9 $$w_{i} (k + 1) = w_{i} (k) + \frac{\mu }{{1/L\sum\limits_{i = 0}^{L - 1} {\left| {z_{i} (k)} \right|^{2} } }}e(k)z_{i} (k)$$

where $k = 0,1, \ldots ,L - 1,$*w*_*i*_(*k*) is the *i*-th weight coefficient at time instant *k*, *µ* is small positive value constant controlling the rate of the adaptive filter convergence, *e*(*k*) is a residual error value between the input and output filter’s signals *e*(*k*) = *d*(*k*) − *y*(*k*). In our case, *e*(*k*) is the EGG signal with attenuated respiration components of the signal. Values of *µ* and *L* were set to 0.0015 and 14, respectively.

### EGG, ExEGG and AfEGG signals extraction

In the present work, the ExEGG signal was extracted by means of adaptive filtering and band pass filtering using the fourth-order Butterworth filter in frequency range 0.015–0.5 Hz. As the reference signal the extracted respiratory signal EDR was used (reconstructed from analysis of the area under QRS complexes). All signals were decimated to 4 Hz before applying adaptive filtering. Different methods were used to obtain the EGG signals. In the first method (classical) the EGG signal was extracted by band pass filtering of resampled to 4 Hz HSECG (EGG4 Hz) signal using the fourth-order Butterworth filter in frequency range 0.015–0.15 Hz. In the second one, the extended ExEGG (EGG4 Hz filtered by means of adaptive filtering) signal was used filtered by means of the fourth-order low pass Butterworth filter with a cutoff frequency of 0.15 Hz, such as result the adaptive filtered EGG (AfEGG) signal is obtained (The flow diagram of signal processing is shown in Figure 3).

### Validation of method, results and discussion

Since the verification of the methods performance as well as established thesis concerning signal extraction from the other organs e.g. duodenum or colon [19] require registration of signals from the surface of these organs, which in turn requires a fairly complex clinical trials (registrations during surgery on patients) it is impossible for the authors of this study, to carry out the test procedures, some of which allow the assessment of the correctness of the proposed algorithm. Two stages of testing have been proposed: first examining the impact of adaptive filtering on the basic parameters of diagnostic EGG tests, i.e. the dominant frequency (DF) and the coefficient of normogastria (NI), second to verify the effectiveness of the detection of additional signals inside the signal EGG. For both testing stages the suitable test signals have been prepared, which were based on actual signals recorded from the surface of the abdomen of patients.

### Filtration influence into EGG signal basic diagnostic parameters

Due to the need to make sure that the proposed method does not change the calculation of the EGG signal diagnostic parameters, a comparison of the most commonly used parameters. DF and NI has been performed to 44 records. Rules for calculating the dominant frequency and the rate of NI normogastria are widely described in the literature [4]. To evaluate the properties of the proposed method, the calculations of the DF and NI factor for the original signal (research). For the calculations used to analyze, the original program for EGG signals processing has been applied. The authors use that software in their research for several years [20–22] (the results generated by the program are verified by comparing the results obtained with the commercial systems used in clinical EGG trials). EGG signals were then delivered to adaptive filtering and recalculation of both DF and NI has been performed. Then to the original EGG signals two sinusoids with frequencies *f*1 = 0.12 Hz and *f*2 = 0.15 Hz have been added. Testing has been conducted for two different values of the amplitudes of the added signals. The amplitude of the added signals was determined by the following relationship:

10 $${\text{A = C}} \times {\text{max(EGG(L:L + 1024)), }}$$

where *A* is the amplitude of the added signal, *C* the correction coefficient equal (respectively *C*1 = 0.1 and *C*2 = 0.2), *L* the randomly selected sample number of the original EGG signal.

The signals prepared in the presented way have been delivered to adaptive filtration and then once again the DF and NI coefficients have been recalculated. Additionally both relative (ε) and absolute (δ) errors between the values of coefficient NI obtained owing to adaptive filtration and the once obtained for originals signals as well as values such maximum and minimum and their SD concerning all the mentioned above errors have been calculated. The results obtained have been placed and presented as Table 1 where EGG stands for original signals EGGAF stands for the same as above but after adaptive filtration and finally (EGG + C1)AF and (EGG + C2)AF are the signals with additional sinusoids added and after their transfer to adaptive filtration.

**Table 1:** Values of NI index and errors

Signal	EGG	EGGAF			(EGG + C1)AF			(EGG + C2)AF		
	NI	NI	ε	δ (%)	NI	ε	δ (%)	NI	ε	δ (%)
S1	0.97	0.97	0.00	0.51	0.95	−0.02	2.55	0.94	−0.03	3.57
S2	0.98	0.98	0.00	0.51	0.99	0.01	1.02	0.98	0.00	0.00
S3	0.85	0.88	0.03	3.51	0.87	0.02	2.92	0.79	−0.06	7.02
S4	0.86	0.83	−0.02	2.89	0.85	−0.01	1.16	0.79	−0.07	8.09
S5	0.76	0.80	0.04	5.13	0.80	0.03	4.49	0.76	0.00	0.00
S6	0.83	0.86	0.03	3.51	0.86	0.03	3.51	0.85	0.02	2.34
S7	0.82	0.84	0.02	2.38	0.81	−0.01	1.19	0.68	−0.14	17.26
S8	0.63	0.70	0.06	10.00	0.67	0.04	6.15	0.62	−0.01	1.54
S9	0.87	0.89	0.02	2.15	0.87	0.00	0.01	0.78	−0.09	10.08
S10	0.89	0.89	0.00	0.00	0.89	0.01	0.70	0.88	−0.01	1.41
S11	0.89	0.87	−0.02	2.11	0.87	−0.02	2.11	0.87	−0.02	2.11
S12	0.81	0.88	0.07	8.46	0.87	0.06	6.92	0.82	0.01	0.77
S13	0.71	0.73	0.02	2.11	0.72	0.00	0.70	0.71	−0.01	0.71
S14	0.70	0.73	0.04	5.03	0.73	0.03	4.32	0.74	0.04	5.75
S15	0.94	0.97	0.03	3.21	0.96	0.02	2.14	0.84	−0.10	10.16
S16	0.82	0.87	0.05	5.49	0.87	0.05	6.10	0.85	0.03	3.66
S17	0.81	0.82	0.00	0.60	0.80	−0.01	1.80	0.67	−0.14	17.37
S18	0.83	0.85	0.02	2.35	0.83	0.00	0.00	0.76	−0.07	8.82
S19	0.92	0.91	−0.01	1.06	0.87	−0.05	5.29	0.78	−0.15	15.87
S20	0.74	0.80	0.06	7.89	0.77	0.03	3.95	0.72	−0.02	2.63
S21	0.55	0.66	0.12	21.69	0.76	0.21	39.18	0.45	−0.09	16.87
S22	0.55	0.64	0.09	16.87	0.68	0.13	23.61	0.53	−0.01	2.41
S23	0.90	0.92	0.02	2.19	0.89	−0.02	1.81	0.93	0.03	3.65
S24	0.87	0.94	0.07	8.33	0.83	−0.04	5.00	0.90	0.03	3.79
S25	0.74	0.76	0.02	2.70	0.84	0.10	12.95	0.69	−0.05	7.43
S26	0.67	0.67	0.00	0.00	0.88	0.21	31.67	0.62	−0.05	6.77
S27	0.88	0.91	0.04	4.00	0.85	-0.02	2.77	0.83	−0.05	5.71
S28	0.87	0.86	-0.02	1.72	0.91	0.04	4.65	0.79	−0.08	9.20
S29	0.85	0.85	0.00	0.59	0.68	−0.17	20.15	0.80	−0.05	5.88
S30	0.87	0.88	0.01	1.15	0.88	0.02	2.02	0.82	−0.04	5.17
S31	0.87	0.89	0.02	2.30	0.86	−0.01	0.99	0.87	0.00	0.00
S32	0.93	0.91	-0.02	2.14	0.79	−0.14	14.85	0.91	−0.02	2.14
S33	0.69	0.77	0.08	11.32	0.85	0.17	24.00	0.35	−0.34	49.06
S34	0.88	0.90	0.03	2.96	0.87	−0.01	0.91	0.81	−0.06	7.41
S35	0.80	0.85	0.05	6.50	0.82	0.02	2.44	0.80	0.00	0.00
S36	0.73	0.79	0.06	8.93	0.86	0.14	18.75	0.73	0.00	0.00
S37	0.78	0.87	0.09	10.97	0.85	0.07	9.03	0.84	0.06	7.10
S38	0.79	0.87	0.09	10.90	0.87	0.08	10.26	0.86	0.07	8.97
S39	0.79	0.82	0.04	4.49	0.82	0.03	3.85	0.74	−0.05	5.77
S40	0.81	0.84	0.04	4.38	0.86	0.06	6.88	0.78	−0.03	3.75
S41	0.80	0.86	0.06	7.44	0.83	0.03	4.13	0.60	−0.20	24.79
S42	0.88	0.86	−0.02	2.24	0.70	−0.18	20.15	0.37	−0.51	58.21
S43	0.66	0.68	0.02	2.97	0.68	0.02	2.97	0.49	−0.18	26.73
S44	0.61	0.74	0.14	22.83	0.64	0.04	6.52	0.43	−0.17	28.26
Max			0.14	22.83		0.21	39.18		0.07	58.21
Min			−0.03	0.00		−0.18	0.00		−0.51	0.00
Mean			0.03	5.19		0.02	7.42		−0.06	9.28
SD			0.04	5.27		0.08	8.94		0.10	12.11

The presented results show that our method does not change the calculated parameters DF and NI and preserve at the same time its main feature i.e. reduces the respiratory component.

### EGG signal additional components detection

**Figure 8:**
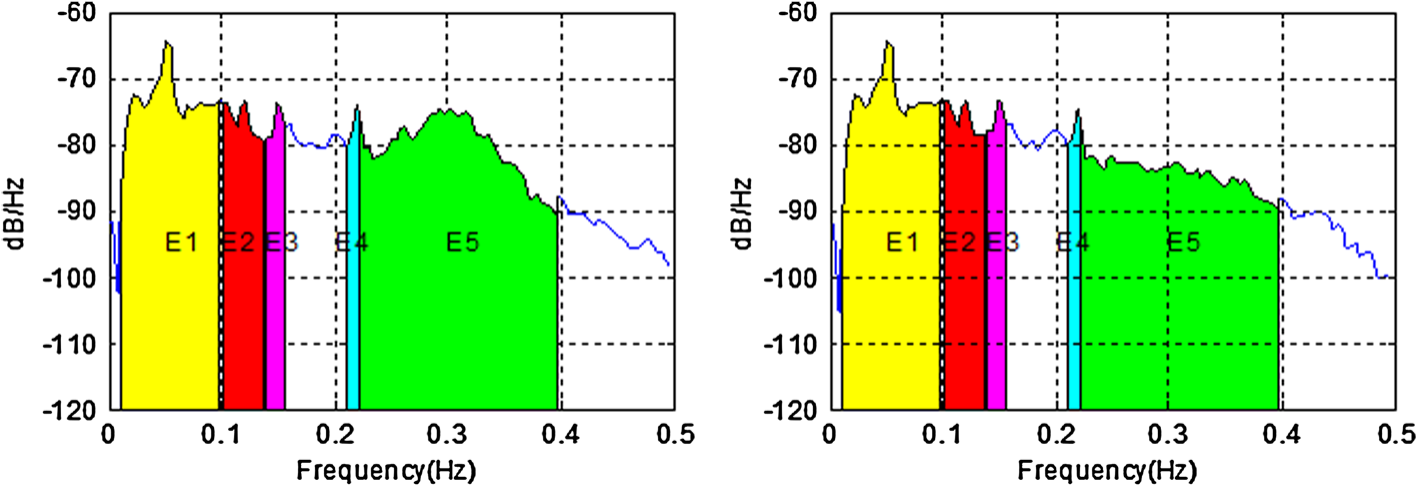
The ranges of calculated area ratios for EGG signal spectra with added sinusoidal components (0.12, 0.15, and 0.22 Hz): spectrum of original EGG signal (*left*) and spectrum of EGG signal after adaptive filtering (*right*).

In order to evaluate the effectiveness of the proposed method detecting signals from the other organs a set containing of test sinusoidal signals added to the original EGG signal with frequencies respectively *f*1 = 0.12 Hz, *f*2 = 0.15 Hz and *f*3 = 0.22 Hz have been prepared. The amplitude of the added signals was 30 μV. Thus prepared signals were subjected to pass through adaptive filtering. Then, the signals were divided into fragments with a length of 1,024 samples (4 min 16 s) and spectrum for each fragment has been determined. The method used to determine the spectra was the periodogram with a Tukey window (*alpha* = 0.25). Based on the obtained spectra the averaged spectrum (roughly equivalent to the designation of the method called overall spectrum for standard analysis EGG [4]) was determined. In order to assess the effectiveness of the proposed method the area under the spectra were calculated in the frequency ranges as follows: (0.01–0.10 Hz), (0.10–0.14 Hz), (0.14–0.16 Hz), 4(0.21–0.23 Hz), (0.22–0.40 Hz) (Figure 8).

**Figure 9:**
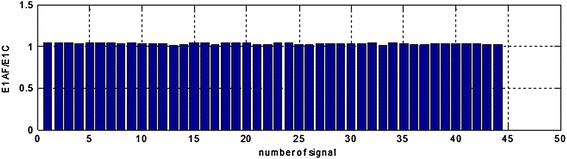
The E1AF/E1C area ratios in range (0.01–0.10 Hz) for all the registered signals.

**Figure 10:**
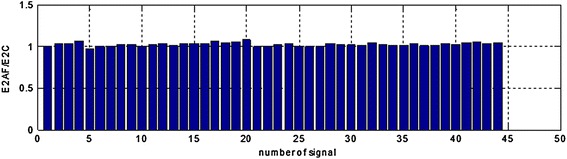
The E2AF/E2C area ratios in range (0.10–0.14 Hz) for all the registered signals.

**Figure 11:**
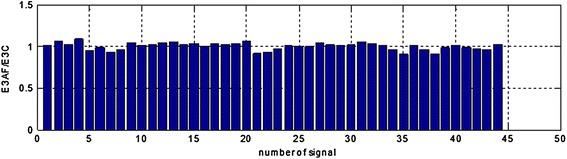
The E3AF/E3C area ratios in range (0.14–0.16 Hz) for all the registered signals.

**Figure 12:**
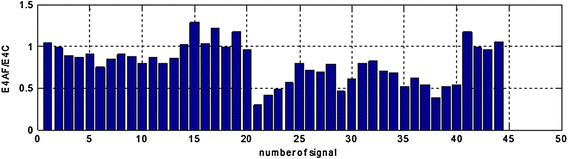
The E4AF/E4C area ratios in range (0.21–0.23 Hz) for all the registered signals.

**Figure 13:**
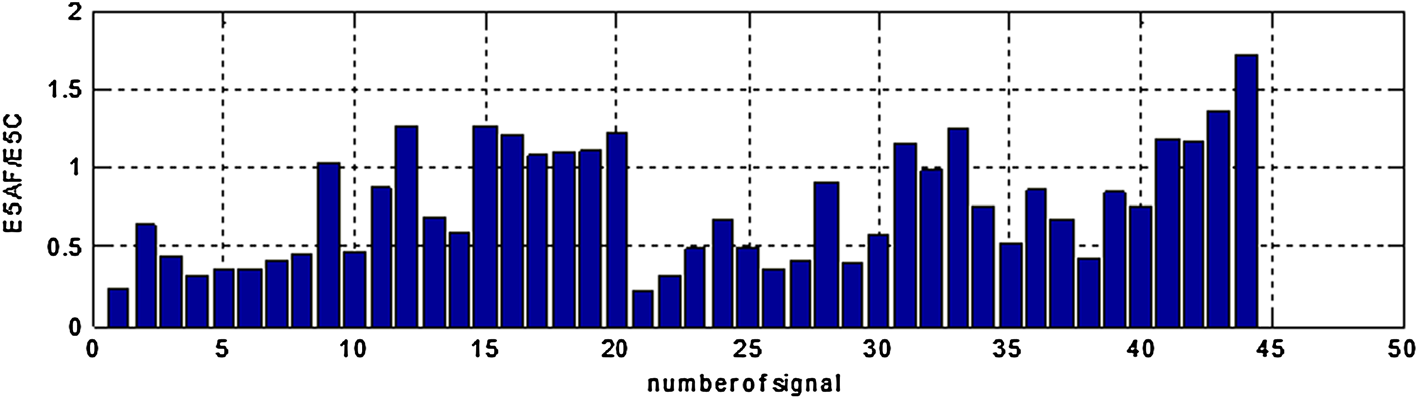
E5AF/E5C area ratios in range (0.22–0.40 Hz) for all the registered signals.

The presented calculations have been performer for both signals with added sinusoidal components and signals with sinusoidal components after adaptive filtration. Next the ratio of the areas concerning EGG signals after adaptive filtration (ExAF) and EGG signals with components added (ExC) in all the particular frequency ranges have been calculated. Results are presented at the following Figures 9, 10, 11, 12, and 13.

**Figure 14:**
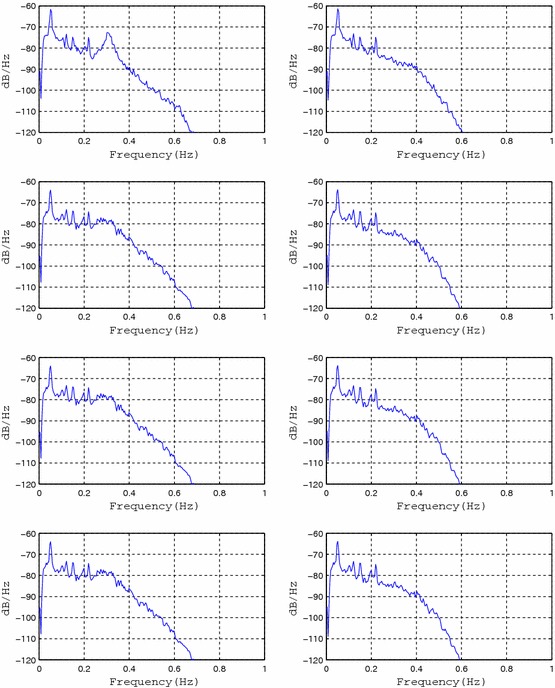
The examples of EGG signals spectra with added components (0.12, 0.15, and 0.22 Hz). Spectra for EGG signals after classical filtering in range (0.015–0.5 Hz) (*left*); spectra of the same EGG signal after adaptive filtering (*right*).

Analysis of the performer tests allows for the following conclusions: suggested method of adaptive filtering does not introduce significant changes inside the typical frequency range concerning EGG signal (i.e. 0.015–0.15 Hz). The mentioned above ratios of the areas under the spectrum equals almost unity. In most analyzed cases the respiratory component (if visible in the EGG signal) is attenuated correctly. This allows the correct retrieval of the searched signal (in our case the signal with frequency 0.22 Hz). The exemplary results are presented as suitable spectra on Figure 14, left part consists spectra of the EGG signals with added components, right part presents the same signals after adaptive filtration. It is easy to notice the influence of adaptive filtering into attenuation of respiratory signal as well as improvement added signals extraction, particularly component consisting of frequency 0.22 Hz.

**Figure 15:**
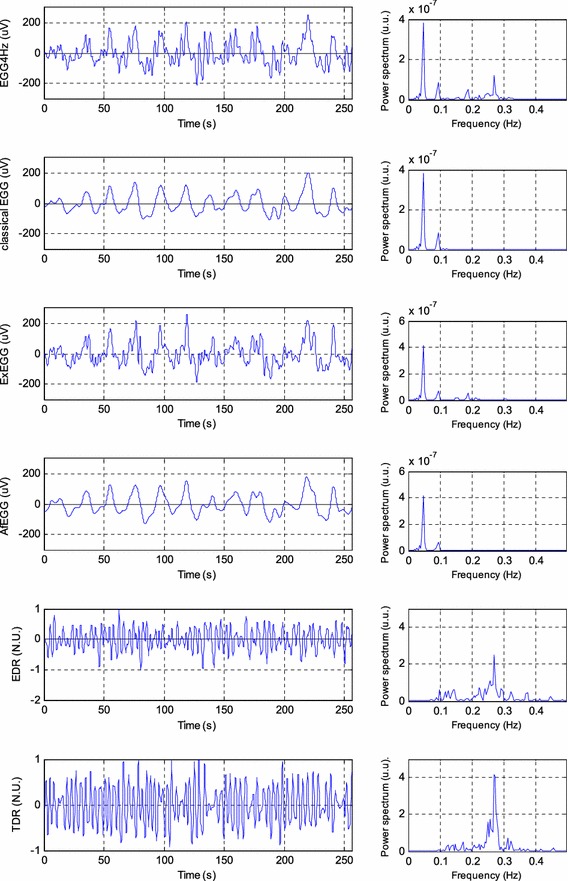
Processed signals and theirs spectra. From top: preprocessed EGG4 Hz (0.15–0.5 Hz) signal (*left*) and its spectrum (*right*), classical EGG (0.015–0.15 Hz) and its spectrum, ExEGG (0.015–0.5 Hz) after adaptive filtering and its spectrum, AfEGG (0.015–0.15 Hz) and its spectrum, derived respiration signal (EDR) and its spectrum, thermistor derived respiration signal (TDR) and its spectrum. The EDR and TDR signals are normalized to the unity.

The component consisting of frequency 0.22 Hz has been chosen as the one not exactly reflecting physiological activity of duodenum and colon. The reason why such a choice has been applied can be explained as follows—that signal is quite near respiratory signals appearing around 0.2 Hz. Such a choice allowed to estimate a correctness of adaptive filtering applied as well as ability to extract components with frequency characteristic overlapping respiratory range. The physiologic respiratory components include also lower frequencies better reflecting the activity of both duodenum and colon. Figure 15 shows the example of the real EGG signal with its spectra consisting of component like 0.18 Hz (very likely reflecting the activity of both duodenum and colon) as well as some respiratory components. This component would be filtered out during classical EGG analysis. Our approach, presented in the paper allows, by using adaptive filtering, removal only respiratory components (signal ExEGG) and recovery of 0.18 Hz component nicely visible in the presented signal.

The proposed method presents an improved selectivity in the suppression of the breathing signal that allows the recording of signals from other organs such as the duodenum or small intestine [23, 24].

## Conclusion

The presented method shows promising prospective for examination of interactions among different systems in human body, such as cardiovascular, digestive, respiratory or neural systems. In this work the possibility of obtaining signals such as EGG, ECG (HRV) and the respiratory signal without any additional sensors or devices is presented. Moreover, these signals may be calculated (extracted) by software only, providing that sampling frequency of signal in the recording device may be slightly increased.

Presented methods show good reconstruction of respiratory signals obtained by the analysis of area under QRS complexes in HSEGG signal. The contribution of low frequency components in the reconstructed respiratory signal obtained by the used method is very low what suggests that this signal includes only respiratory components.

The respiratory signal (EDR) serves well as the reference signal in adaptive filtering for attenuating the respiratory components in signals. An analysis of spectra of reconstructed signals confirms good efficiency of attenuating the respiratory components in EGG signals by means of the proposed adaptive filtering method.

Efficiency of attenuating the respiratory components depends on parameters of adaptive filter. Because investigations presented in this paper were made on relatively small number of cases (44), the parameters *μ* and *L* may require some corrections and future investigations. It is very likely that the presented method of the HSEGG signal acquisition allows both acquisition and processing of signals from inner organs of digestive system such as the duodenum and colon, but eventual confirmation of this conclusion requires future investigation and close cooperation with gastrointestinal specialists.

## Abbreviations

HSEGG: high sampling frequency electrogastrographic signal
NSDECG: non-standard derivation electrocardiographic signal
EGG: electrogastrographic signal, electrogastrography, electrogastrogram
ECG: electrocardiographic signal
EDR: electrocardiographic derived respiration signal
DCT: discrete cosine transform
HRV: heart rate variability
ExEGG: extended EGG signal
TDR: thermistor derived respiration signal
